# Dynamics of B-Cell Populations in CSF and Blood in Patients Treated with a Combination of Rituximab and Mitoxantrone

**DOI:** 10.1155/2013/748127

**Published:** 2013-09-10

**Authors:** Evgeniy Evdoshenko, Alexey Maslyanskiy, Sergey Lapin, Leonid Zaslavsky, Ruth Dobson, Areg Totolian, Alexander Skoromets, Amit Bar-Or

**Affiliations:** ^1^St. Petersburg's Center of MS and Autoimmune Diseases, City Hospital N31, Saint Petersburg, Russia; ^2^Department of Rheumatology, Federal Clinical Center of Heart, Blood and Endocrinology, Saint Petersburg, Russia; ^3^Laboratory of Autoimmune Diagnostics, Saint-Petersburg Pavlov Medical University, Saint Petersburg, Russia; ^4^Department of Neurology, Leningrad Region Hospital, Saint Petersburg, Russia; ^5^Neuroimmunology Group, Blizard Institute, Great Britain, UK; ^6^Laboratory of Immunology, Pasteur Institute for Microbiology and Epidemiology, Saint Petersburg, Russia; ^7^Department of Neurology, Saint-Petersburg Pavlov Medical University, Saint Petersburg, Russia; ^8^Department of Neurology and Neurosurgery and Department of Microbiology and Immunology, McGill University, Montreal, QC, Canada

## Abstract

*Background*. Mitoxantrone (MTX) and Rituximab (RTX) are successfully used for treatment of multiple sclerosis (MS) and can be combined to increase efficacy. *Objective*. We used MTX, RTX, and methylprednisolone in a single combined regiment and observed patients prospectively. *Methods*. We present results of observational pilot study of combined therapy of RTX and MTX in 28 patients with active MS. Therapeutic protocol consisted of two infusions within 14 days. First infusion was 1000 mg methylprednisolone (MP) IV, 1000 mg RTX IV, and 20 mg MTX IV. On day 14, 1000 mg MP IV and 1000 mg RTX IV were given. Patients were followed prospectively from 12 to 48 months. *Results and Conclusion*. There were no relapses among all 28 patients during the observation period. B-cell depletion of CD19+ and CD19+/CD27+ memory B-cell subpopulation in both compartments was confirmed in all patients at 6 months. We found a more rapid reconstitution of B cells in the CSF than in the peripheral blood and longstanding depression of CD19+CD27+ memory B-cell. *Conclusion*. Effectiveness of combined regimen of RTX and MTX could be related to longstanding depletion of CD19+CD27+ memory B-cell subset.

## 1. Introduction

Multiple sclerosis (MS) is an autoimmune inflammatory disease of the CNS, characterized by focal demyelination, loss of axons, and gliosis that result in neurological symptoms. While our understanding of MS immunopathology continues to improve, the underlying etiology of the disorder remains unclear.

The overall efficacy of traditional MS treatments (beta-interferon, glatiramer acetate) is limited, and these drugs are widely accepted to have a relatively small effect on disease activity [1, 2]. More powerful approaches to disease modification in MS include the so-called “biological therapies” or monoclonal antibodies (natalizumab, alemtuzumab, daclizumab, rituximab, and ocrelizumab) and cytotoxic drugs (cladribine and mitoxantrone) [3–6]. The use of these therapies is limited by concerns regarding potential side effects, including an increased risk of infection and a theoretical increase in the lifetime risk of malignancy. It is thought that at least some of these risks increase as the cumulative dose of the drug increases [7].

Clinical trials of the anti-CD20 monoclonal antibody rituximab (RTX) in rheumatoid arthritis, systemic connective tissue diseases, and ANCA-associated vasculitides have reinforced its position as one of the leading potential therapeutic options in a range of autoimmune diseases [3, 8–11]. In MS, CD20+ B cells are rapidly becoming recognized as a valid therapeutic target, partly due to an increasing interest in their role in MS pathogenesis. The role of B cells in MS appears to be wide ranging and pervasive and includes the production of autoantibodies, antigen presentation to T cells, proinflammatory cytokine release, and tertiary lymphoid tissue formation in CNS of MS patients [12]. 

Studies examining the efficacy of RTX as a therapy in MS are limited. The largest and most important study to date is Helping to Evaluate Rituxan (rituximab) in Relapsing-Remitting Multiple Sclerosis (HERMES). Results demonstrated the ability of RTX to substantially control the focal inflammatory process in MS based on both MRI and clinical data. However, RTX did not completely suppress relapses—10 patients (14.5% of patients) experienced a clinical relapse during the first 24 weeks of the study [13]. Besides the inability of RTX to cross the blood-brain barrier, the limited efficacy of RTX monotherapy could also be explained by the capability of B cells to survive in certain tissues—referred to as “privileged sites.” This phenomenon has been seen in rheumatoid arthritis; pathological B cells found in the synovial lining of joints were decreased in number but not eliminated by RTX treatment [14, 15]. 

Mitoxantrone (MTX) is a topoisomerase II inhibitor that is commonly used in the treatment of MS. Its clinical efficacy has been proven in a number of controlled studies [16, 17]. In animal models of MS, MTX was found to be tenfold more potent when compared to cyclophosphamide [18]. The effect of MTX may be partly due to its ability to induce B-cell apoptosis. It appears that the subpopulation that is most sensitive to this effect is the CD19^+^/CD27+ memory B cells. Additional clinical studies have shown correlations between CD19^+^CD27^+^ memory B-cell pool depletion and therapy effectiveness. [19–21]. 

There are some lines of evidence that the combination of RTX and cytotoxic therapies could result in a more profound reduction in B-cell number. The combination of MTX and RTX forms part of the FCM-R regimen (fludarabine, cyclophosphamide, MTX, and RTX), which is used in the treatment of B-cell neoplasms with acceptable tolerability [22]. It appears that RTX and MTX could target B cells through different mechanisms. Therefore, we hypothesized that the combination of RTX with MTX may be effective in treatment of MS with high disease activity. 

In this paper, we present the results of an observational pilot study of 28 MS patients who received a single course of combined therapy with RTX-MTX together with methylprednisolone (MP) and were followed clinically and immunologically with a maximal follow-up of 48 months. This study still provides an important insight into immunotherapy in MS.

## 2. Material and Methods

### 2.1. Patient Groups and Study Protocol

We assessed the efficacy of a single course of combined immunosuppressive therapy consisting of RTX and MTX together with methylprednisolone (MP) in MS. 28 patients with clinically definite MS (MacDonald 2005 criteria [23]) aged 18 to 55 who had at least one relapse in the 6 months prior to enrolment were recruited. All patients provided informed consent prior to study enrolment, and the study was approved by the local ethics committee.

The study group consisted of 8 patients resistant to standard therapy (beta-interferons, glatiramer acetate) and 8 patients who were resistant to different therapeutic approaches including bone marrow transplantation and 12 therapy naïve patients with very active MS. Disease modifying agents were withdrawn 1 month prior to any treatment in this study. The clinical details of the study participants are summarised in Table 1. 

The therapeutic protocol consisted of two infusions 14 days apart and was performed as an inpatient during two separate hospital admissions to a specialised neurological department. On the first day, 1000 mg methylprednisolone (MP) IV was administered. On the second day, 1000 mg MP IV, 1000 mg RTX IV, and 20 mg MTX IV were given consecutively. Premedication with 1 g acetaminophen (paracetamol) and 20 mg diphenhydramine (piriton) IV was given 30 minutes prior to RTX infusion. On the third day, 1000 mg MP IV was administered. The patients were readmitted on day 14 when 1000 mg MP IV and 1000 mg RTX IV were administered. 

For the initial three weeks, we checked complete blood counts and urinalysis every third day. Clinical and laboratory data as well as MRI examination were performed at several fixed time-points during the first year: 6 months, 9 months, and 12 months. After the first year of followup, patients were observed clinically at intervals of 6 months. At the date of paper preparation, the minimum duration of followup was 12 months and maximum observation period was 48 months. 

The primary clinical endpoint was the tolerability of the combined regimen, and the primary imaging endpoint was the reduction in number of gadolinium-enhancing MRI lesions. 

An aim of the study was to determine the changes in clinical disease measures, in the dynamics of B-cell populations cerebrospinal fluid (CSF), and in peripheral blood (PB). As there was no placebo arm, clinical examinations were unblinded and performed for safety rather than efficacy. 

### 2.2. Clinical and Magnetic Resonance Imaging Examinations

Before treatment, all patients underwent an extended neurological examination with formalised clinical scales (EDSS and MSFC) and an MRI. All patients were screened for viral hepatitis (B,C), HIV-1, and syphilis and had echocardiography and a chest X-ray performed. Blood and CSF were sampled 1 day prior to treatment. 

The safety of the combination of RTX and MTX was determined by infusion-associated adverse events (AEs), drug-related AE, and infection-associated AE according to the National Cancer Institute (NCI) Common Toxicity Criteria (CTC) version 3.0 grading system over the study period [24, 25]. 

MRI examinations were performed with 1.5 T MRI apparatus (Siemens AG, Germany). Imaging endpoints were the change from baseline in the total number of gadolinium- (Gd-enhancing) T1 lesions, the total number of new T2 lesions, and the number of lesions changing from T2 to T1. MRI section thickness was 1 mm, and lesions greater than 3 mm in two dimensions were scored. 

### 2.3. Flow Cytometry and Other Immunological Tests

Peripheral blood was taken on the day of lumbar puncture and examined using flow cytometry for the expression of lymphocyte markers CD3, 4, 8, 16, 56, 19, 27, HLADR, and CD4/25. Samples of CSF were taken by lumbar puncture. Aliquots of 5 mL CSF were sent for flow cytometry studies. The cells were spun down with careful centrifugation (1000 G), and 1 mL of cell pellet was taken for analysis. In all cases, samples were analysed within 4 hours of lumbar puncture.

A 50 mL aliquot of CSF was sent for immunological studies including the detection of IgG oligoclonal bands by isoelectric focusing and immunoblotting in paired CSF and serum samples, according to established methods [26].

## 3. Results

### 3.1. Safety

AEs were systematically examined during the study in all enrolled patients. An immediate infusion reaction of a urticarial rash and weakness developed in 1 patient and resolved spontaneously within 24 hours following the first infusion. This did not recur at the time of the second rituximab infusion (Table 2). 

**Figure 1:**
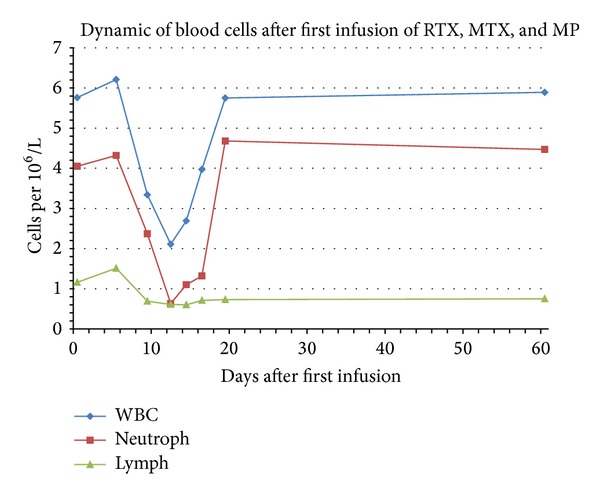
Dynamic of mean values of white blood cells (WBC), neutrophils (Neutroph), and lymphocytes (Lymph) after first infusion of rituximab with mitoxantrone and intravenous methylprednisolone. *x*-axis represents days after infusion.

A transient neutropenia of less than 1500 cells per microlitre was found in 100% (28/28) of patients but completely resolved in all patients by day 19 since the first infusion. This represents an AE that is recognized as grade 1–3 toxicity. In 46% (13/28) of patients, neutrophil counts were less than 500 cells per microlitre—that is, grade 4 toxicity. Detailed data on leukocyte and neutrophil counts is presented in Figure 1. This neutropenia was not complicated by infection or neutropenic fever, and spontaneous resolution was noted in all patients within 5–7 days. Malaise and fatigue were associated with neutropenia in all patients. Other noninfectious AEs are presented in Table 2.

Upper respiratory tract infections including the common cold and nasopharyngitis were most frequent during the first year of observation and were seen in 13 (46%) patients. In all cases, infections were mild and resolved without antibiotic therapy. 

### 3.2. Clinical and Imaging Data

**Figure 2:**
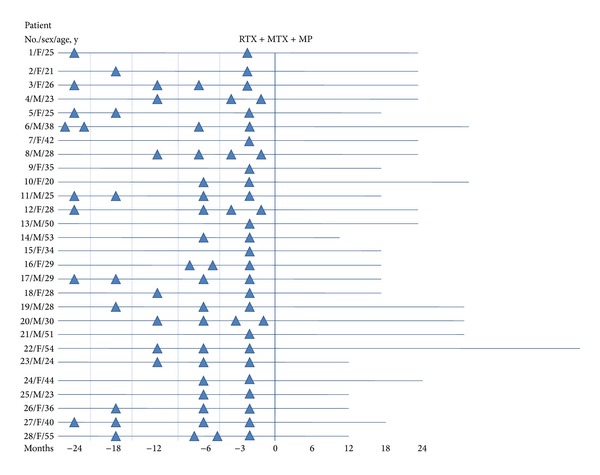
Number of relapses and period of observation before and after treatment. Month 0: start of the treatment; ▲: relapse.

There were no relapses amongst patients receiving the combined therapy during the period of observation (Figure 2). 

**Figure 3:**
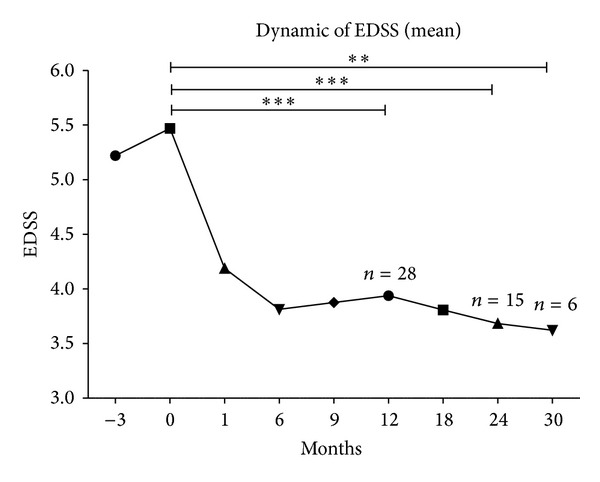
Dynamic of EDSS (mean) for a period observation of 30 months. Significant decrease in the EDSS score from 5.1 ± 1.7 before treatment to 3.5 ± 2.0 (*P* < 0.0001) at 12 months after treatment initiation in 28 patients and a decrease in the EDSS score from 5.1 ± 1.7 before treatment to 3.5 ± 2.0 (*P* < 0.0001) at 24 months after treatment in 15 patients. **P < 0.001; ***P < 0.0001.

We found that treatment was associated with a significant decrease in the EDSS from 5.1 ± 1.7 to 3.5 ± 2.0 (*P* < 0.0001) in those patients followed up for 24 months (Figure 3). In 89% of patients, a persistent decrease in the EDSS score was seen, without any maintenance therapy. In 3/8 patients who were previously treated with bone marrow transplantation, a gradual increase of EDSS was seen (mean 0.5 point) by 6, 12, and 18 months of observation. 

The baseline MRI examination was performed during the 3 months prior to the start of treatment. 89.3% (25/28) of patients had Gd-enhancing MRI lesions. The mean number of Gd-enhancing lesions was 4.43 ± 2.90 (range 0–10). All patients manifested new T2 lesions within the 3 months prior to therapy. The mean count of T2 lesions was 42 ± 31 (range 4–133), and the mean count of T1 lesions was 15 ± 12 (range 0–47). 

**Figure 4:**
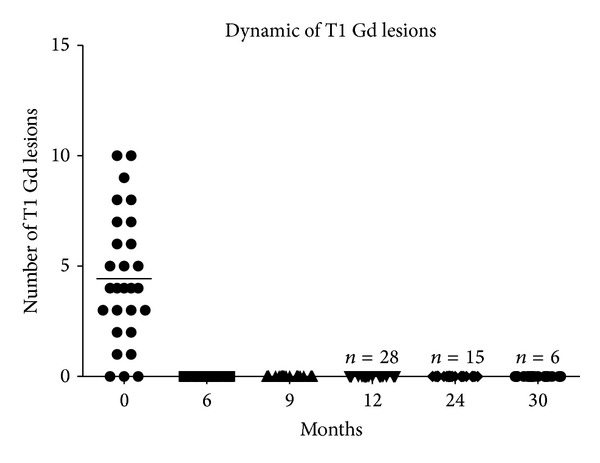
Dynamic of Gd-enhanced lesions after therapy with rituximab with mitoxantrone and intravenous methylprednisolone during observation period. Along the period of observation after combined therapy, there were neither Gd-enhancing MRI lesions nor new T2 lesions in any of the study patients.

After 12 months of followup, the mean number of T2 lesions was 42 ± 32 (range 4–133) and T1 lesions 15 ± 12 (range 0–47) (Figure 4). During the study followup, no patients developed either Gd-enhancing or new T2 lesions. 

There was a positive correlation between the number of Gd-enhancing lesions prior to treatment and the decrease in EDSS. A more pronounced decrease in EDSS at 6 months was seen in those patients who had more Gd-enhancing lesions at baseline (*r* = 0.42, *P* = 0.025). 

### 3.3. Immunology

**Figure 5:**
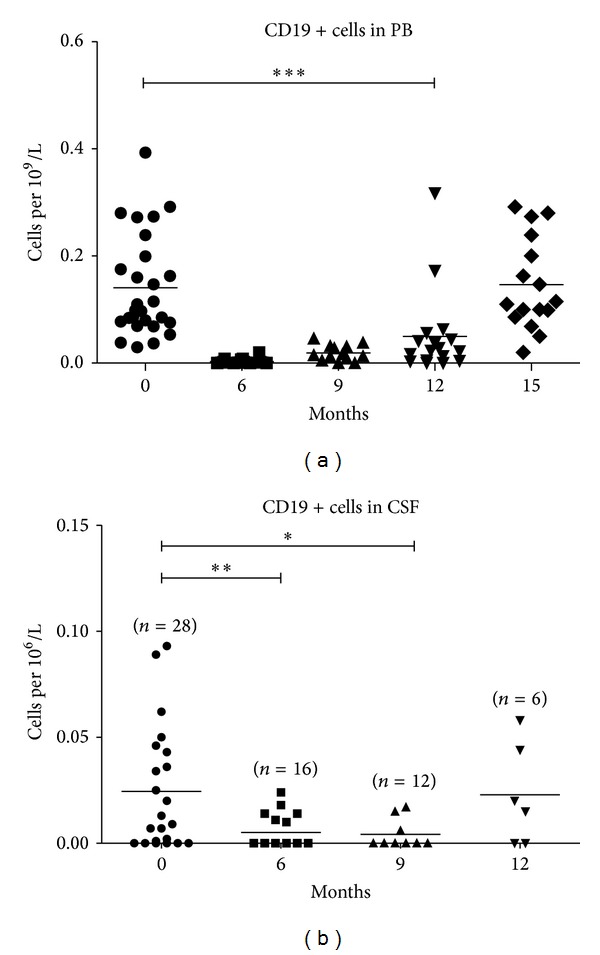
Dynamic of CD19+ cells in peripheral blood (a) and CSF (b) after treatment. *P < 0.05;  **P < 0.001; ***P < 0.0001.

**Figure 6:**
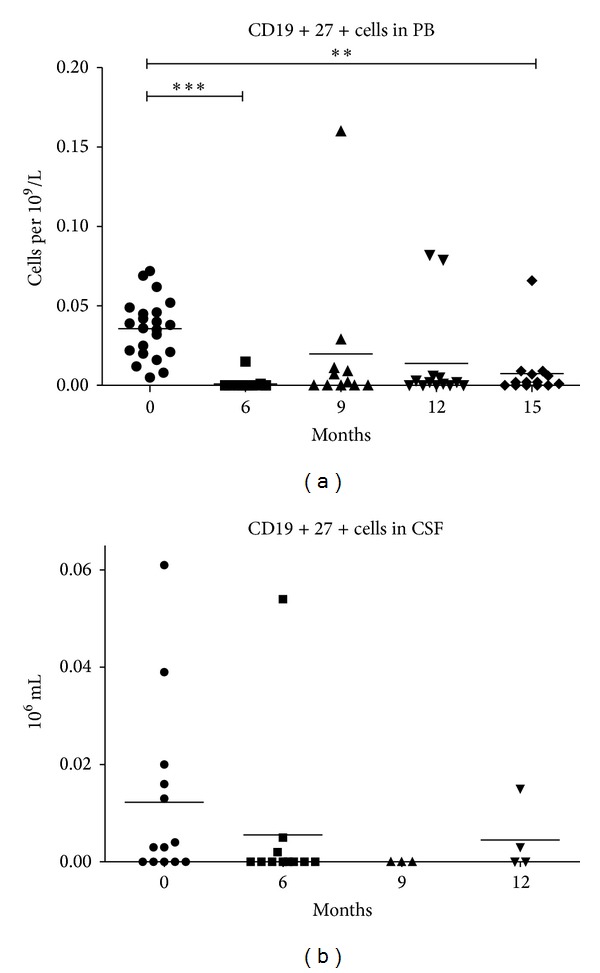
Dynamic of CD19+CD27+ memory B cells in peripheral blood (a) and CSF (b) after treatment. ***P < 0.0001; **P = 0.0059.

We performed serial measurements of CD3-CD19+ B-cell and CD3-CD19+CD27+ memory B-cell subpopulations in peripheral blood and CSF. B-cell depletion (>95% reduction from baseline) in peripheral blood was confirmed in all patients at 6 months (Figure 5(a)). At 9 months, B cells reappeared in the peripheral blood (mean 0.018 ± 0.014× 10 ∗ 9 cells per liter) and expanded to 0.049 ± 0.080× 10 ∗ 9 cells per liter. In the majority of the patients, complete restoration of the number of B cells in peripheral blood occurred at 15 months after treatment. However, the number of CD27+ memory B cells was still significantly decreased compared to baseline (Figure 6(a)). 

The number of B cells in peripheral blood fell over 150 times, while in the CSF the decrease was less pronounced and resulted in an approximate 5-fold reduction (Figure 5(b)). In the CSF, B cells dropped from 0.024 ± 0.028× 10 ∗ 6 cells per liter to 0.005 ± 0.008× 10 ∗ 6 cells per liter (*P* = 0.0034). By 12 months, B cells in the CSF had reached their initial levels of 0.022 ± 0.023× 10 ∗ 6 cells per liter (*P* = 0.687).

We found a more rapid reconstitution of B cells in the CSF than in the peripheral blood. By 9 months, B cells in the peripheral blood were still significantly lower (*P* = 0.001) than prior to therapy, whilst in the CSF, the number of B cells had returned to the levels observed prior to treatment (Figure 5). 

Oligoclonal IgG bands in CSF samples were tested before therapy and at 12 months after therapy and were found in all patients. The frequency and intensity of oligoclonal IgG bands were unchanged at 12 months. 

## 4. Discussion

The rationale for our proposed treatment strategy is the general finding that “biologic” agents are more effective if used in combination with immunosuppressive or antiproliferative drugs. In the treatment of rheumatoid arthritis or system lupus erythematosis, RTX monotherapy is significantly less potent than RTX used in combination with another cytostatic drug such as cyclophosphamide or methotrexate [8]. 

We treated 28 patients with a single therapeutic course of RTX-MTX-MP in combination and followed these patients for more than 1 year. All patients had very high MS activity at the time of enrolment in this study. 

The treatment regimen was generally well tolerated and any immediate infusion reactions observed were mild. The unexpected finding in all patients treated with the combined therapy of RTX-MTX-MP was the high frequency of early neutropenia noticed by days 7–12. In over 40% of patients, the absolute neutrophil number was less than 500 cells per microliter. Neutropenia was characterized by fast recovery, taking less than 7 days, and the neutropaenia did not require treatment with colony-stimulating factors and/or antibiotics. There were no infection-related AEs associated with neutropaenia.

Neutropenia is a recognised rare adverse effect of RTX therapy and is classified into early onset and late onset forms. The latter is more clinically relevant and typically occurs 2 to 4 months following RTX administration [27]. According to the data on hematological complications of RTX monotherapy, late onset RTX-induced neutropenia is seen in 6% of patients treated with a single course of the drug [28]. Cytotoxic drugs such as cyclophosphamide, which have been administered together with RTX in the treatment of systemic lupus erythematosis and other rheumatological diseases, have not been found to increase the frequency of either early or late onset RTX-induced neutropenia [20]. 

Published data suggests that late onset neutropenia could be related to an excess of T-lymphocyte populations which express and secrete large amounts of Fas and Fas Ligand (FasL) leading to the apoptosis of mature neutrophils and the production of antineutrophil autoantibodies [29–31]. Another possible cause of neutropenia in RTX-treated patients is associated with the very high levels of BAFF, a strong stimulator of B-cell recovery, that can be seen in patients after B-cell depletion. These high levels could, in theory, promote B-cell lymphopoiesis in the bone marrow at the expense of normal granulopoiesis [32].

The dose of MTX typically recommended for monotherapy for MS (12 mg/m^2^) induces transient neutropenia in approximately one-third (27%) of patients [33]. When administered as a monotherapy, MTX-induced neutropenia usually peaks at 7–14 days and resolves by 21 days following treatment initiation. 

Our patients were followed clinically throughout the observation period which varied from 12 to 48 months. We confirmed suppression of MS activity and remission in 89% of patients that was sustained during followup. The absence of Gd-enhancing and new T2 lesions on repeated MRI suggests a profound decrease of inflammatory activity in these previously highly active MS patients. In most of the patients, clinical course of MS after single course of the combined therapy did not require any maintenance therapy during observation at the moment of the last data collection.

We noted B-cell depletion in peripheral blood which persisted for up to 15 months. In general, our data on the dynamics of B-cell counts is compatible with the results of the HERMES study. In the HERMES study, it was shown that RTX monotherapy induced a rapid depletion of CD19+ peripheral B lymphocytes from 2 weeks after treatment until 24 weeks. By week 48, CD19+ cells had returned to 30.7% of baseline values [13]. The number of CD27+ memory B cells in peripheral blood was still significantly decreased compared to baseline. We have also shown that in the CSF B cells reappeared at 9 months, and there was a preservation of oligoclonal IgG bands at 1 year that probably reflects propagation of intrathecal B cells.

The high frequency of neutropenia and long-term decrease in CD27+ memory B cells in our patients may reflect a biological synergism of the proposed combination of RTX and MTX with MP. Synergistic drug combinations of few relatively “strong” medications can potentially decrease the cumulative dose of any one medication and thus reduce some treatment associated risks. Such a combination could possibly be reserved as an escalation opportunity for patients with particularly active disease and poor prognostic factors [34], or as a preferable alternative to other induction approaches such as alemtuzumab [35], cladribine [4], high-dose cyclophosphamide (RevImmune) [36], and bone marrow transplantation.

## Figures and Tables

**Table 1:** Summary of clinical data of patients under study.

Patient no./sex/age, yrs	Type of MS	Time since initial symptoms, yrs	Time since diagnosis, yrs	MS therapy in previous 5 yrs	EDSS at baseline	Relapses in previous 2 yrs	MRI findings (within 6 months before treatment) T2/T1/T1Gd	OCB
1/F/25	RR	3	1	None	3.0	3	34/22/5	+
2/F/21	RR	3	2	None	2.0	2	48/9/3	+
3/F/26	SP	4	3	INF*β*-1b 250 *μ*g	6.0	4	35/11/3	+
4/M/23	RR	2	2	INF*β*-1a 30 *μ*g	3.0	3	24/8/2	+
5/F/25	RR	2	2	INF*β*-1b 250 *μ*g	4.0	3	53/18/3	+
6/M/38	PP	12	10	GA Auto-HSCT	6.0	4	38/20/0	+
7/F/42	RR	6	6	Auto-HSCT	2.0	1	38/15/5	−
8/M/28	SP	10	10	INF*β*-1b 250 *μ*g	7.5	4	31/12/10	+
9/F/35	SP	12	10	Auto-HSCT	6.0	1	38/23/0	+
10/F/20	RR	2	2	INF*β*-1b 250 *μ*g	4.0	2	133/34/7	+
11/M/25	RR	3	3	None	4.5	4	23/6/4	+
12/F/28	RR	4	3	INF*β*-1b 250 *μ*g Mitoxantrone	5.0	4	24/7/8	+
13/M/50	RR	10	5	None	4.0	1	40/21/5	+
14/M/53	RR	1	0	GA	6.5	2	24/0/10	+
15/F/34	RR	6	4	GA	4.0	1	30/10/4	+
16/F/29	RR	4	2	None	6.0	3	27/8/4	+
17/M/29	RR	15	14	None	6.0	4	92/47/6	+
18/F/28	RR	3	2	INF*β*-1b 250 *μ*g	4.0	2	33/8/1	+
19/M/28	SP	4	2	None	6.0	3	24/8/9	+
20/M/30	SP	3	3	GA mitoxantrone	9.0	4	22/13/7	+
21/M/51	SP	12	10	INF*β*-1b 250 *μ*g	6.0	1	32/20/0	+
22/F/54	SP	13	10	GA mitoxantrone	6.5	3	28/19/6	+
23/M/24	RR	1	1	None	3.0	3	130/40/2	+
24/F/44	SP	6	5	None	6.0	2	36/3/1	+
25/M/23	RR			None	4.0	2	12/0/4	+
26/F/36	RR	4	4	None	6.0	3	4/1/4	+
27/F/40	RR	4	3	None	5.5	4	89/23/3	+
28/F/55	SP	3	3	Mitoxantrone	8.0	4	22/2/8	+

RR: relapsing-remitting; PP: primary progressive; SP: secondary progressive; INF*β*: interferon beta; GA: glatiramer acetate; Auto-HSCT: autohematopoietic stem cells transplantation; T2(PD): hyperintense lesions; T1: hypointense lesions; T1Gd: gadolinium-enhanced lesions; OCB: oligoclonal bands.

**Table 2:** Adverse events in study population according to the common toxicity criteria, version 3.0.

Events	Patients *n* (%)
Drug-related events affecting	
Headache	5 (17.8%)
Nausea	3 (10.7%)
Fatigue	10 (35.7%)
Throat irritation	1 (3.5%)
Infusion-associated event—number of patients (%)***	
First infusion at w 0	1 (3.5%)
Second infusion at w 2	0
Specific infection-associated event—number of patients (%)	
Nasopharyngitis	11 (39.2%)
Upper respiratory tract infection	2 (15.3%)
Urinary tract infection	1 (3.5%)
Sinusitis	1 (3.5%)
Common toxicity criteria	
Grade 1	28 (100%)
Grade 2	24 (86%)
Grade 3	16 (57%)
Grade 4	13 (46%*)
Grade 5	0
Serious adverse event*	0 (0%)

*All cases presented with transient neutropenia (less than 500 cell/mL) which resolved spontaneously by day 19.

**Serious adverse events were defined as life threatening, resulting in death, requiring prolonged inpatient hospitalization, resulting in a congenital anomaly or malignant condition, or requiring surgical intervention to prevent one of these outcomes.

**Infusion-associated event included any adverse event occurring during or within 24 hours after rituximab and mitoxantrone infusion in all patients.
